# Early postoperative laboratory parameters are predictive of initial treatment failure in acute septic arthritis of the knee and shoulder joint

**DOI:** 10.1038/s41598-023-35384-1

**Published:** 2023-05-20

**Authors:** Jennifer Straub, Marie-Therese Lingitz, Sebastian Apprich, Kevin Staats, Reinhard Windhager, Christoph Böhler

**Affiliations:** Department of Orthopaedics and Trauma Surgery, Division of Orthopaedics, Medical University of Vienna, Währinger Gürtel 18-20, 1090 Vienna, Austria

**Keywords:** Risk factors, Musculoskeletal system, Orthopaedics

## Abstract

Septic arthritis is an orthopedic emergency potentially causing irreversible joint damage. However, the predictive value of potential risk factors such as early postoperative laboratory parameters remains uncertain. We investigated risk factors for initial surgical treatment failure using data from 249 patients (194 knees, 55 shoulders) treated for acute septic arthritis between 2003 and 2018. Necessity for further surgical intervention was defined as primary outcome. Demographic data, medical history, initial and postoperative laboratory parameters, Charlson Comorbidity Index (CCI), and Kellgren and Lawrence classification were collected. Two scoring systems were developed as tools for failure risk estimation after initial surgical irrigation and debridement. More than one intervention was necessary in 26.1% of cases. Treatment failure occurred significantly more often for those with longer symptom duration (p = 0.003), higher CCI grades (p = 0.027), Kellgren-Lawrence grade IV (p = 0.013), shoulder arthroscopy (p = 0.010), positive bacterial culture results (p < 0.001), slow postoperative CRP decline until day three (p = 0.032) and five (p = 0.015), reduced WBC-decline (p = 0.008), and lower hemoglobin (p < 0.001). Scores for third and fifth postoperative day achieved AUCs of 0.80 and 0.85, respectively. This study identified risk factors for treatment failure in patients with septic arthritis, suggesting that early postoperative laboratory parameters can guide further treatment.

## Introduction

Septic arthritis presents an orthopedic emergency with high mortality, and if treated delayed it can cause irreversible bone and cartilage destruction^1–3^. The annual incidence ranges from 4 to 10 per 100.000, with an increasing trend over the last two decades^4–7^. The knee is most commonly affected followed by the shoulder, and infections are either caused hematogenous, iatrogenous, or posttraumatic^5,7,8^. Several studies have identified risk factors for septic arthritis of native joints such as rheumatoid arthritis, osteoarthritis, immunosuppression, concurrent infection, diabetes mellitus, or intraarticular corticosteroid injections^1,9–11^.

Diagnosis is based on patient history, physical examination, laboratory values, imaging, and synovial fluid aspiration. Bloodwork ideally includes C-reactive protein (CRP), erythrocyte sedimentation rate (ESR), a complete blood cell count, metabolic panel, and blood cultures before initiation of antibiotic treatment. Further, arthrocentesis with synovial fluid white cell count (WCC), gram strain, crystal analysis, PCR, and cultures are performed as standard^2,12,13^. The most frequent pathogen in synovial fluid cultures is *Staphylococcus aureus*, followed by streptococcal species^8,14,15^.

Patients with septic arthritis are usually treated with irrigation and debridement either through arthroscopy or open arthrotomy, in combination with culture-adapted antibiotic therapy, but currently no joint specific treatment algorithms exist, and study results are equivocal regarding optimal surgical techniques^2,7,12,13,16,17^. Failure rates of a single intervention remain high, and in 11 to 40% of all patients more than one debridement is needed to eradicate the infection^2,3,7,18–22^. So far, few studies have addressed the risk factors for failure of a single surgical debridement^19–24^. To our knowledge, no study has yet analyzed the time courses of laboratory values during treatment of septic arthritis and their influence on reoperation rates.

Therefore, the current study aimed to identify clinical as well as early serological risk factors for treatment failure and additionally to develop predictive models for prognosis of single surgical intervention failure.

## Materials and methods

### Patients

**Figure 1:**
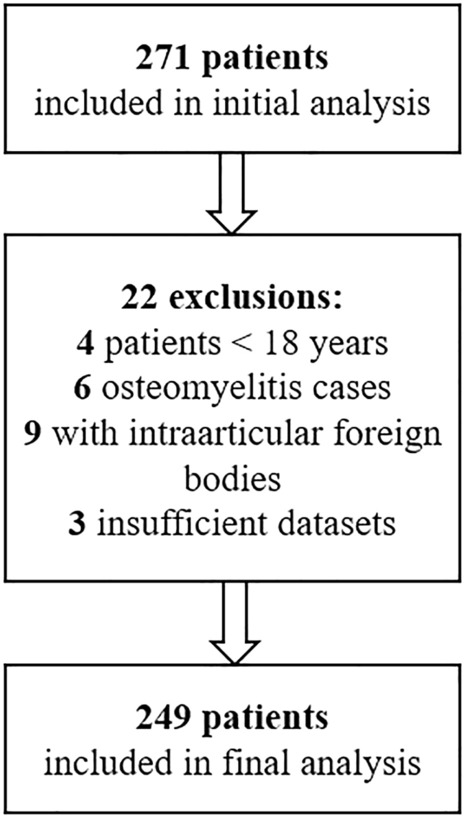
Flow-chart diagram of the exclusion process.

In this case–control study data was collected retrospectively from all patients treated with arthroscopy or arthrotomy for septic monoarthritis of the native knee or shoulder joint at our institution between 2003 and 2018. We excluded patients with a diagnosis of osteomyelitis or periprosthetic infections, intra-articular foreign bodies, and all patients younger than 18 years (Fig. 1). Initial diagnosis based on clinical presentation, inflammatory markers, imaging, and joint aspiration, and had to be confirmed by a positive culture of the joint fluid or by histopathological examination. 249 patients were included in the final analysis.

### Demographics

We collected demographic data as presented in Table 1, and pre- and postoperative laboratory findings as presented in Table 2. The Charlson Comorbidity Index (CCI) was used to summarize comorbidities. Further, surgery type (arthroscopy or arthrotomy) and duration were recorded. Severity of osteoarthritis was graded via the Kellgren and Lawrence classification based on available radiographs^25^, and the Gächter classification was used to grade intra-articular inflammation^26^.

**Table 1:** Patient characteristics and surgery types described by mean (SD) or absolute numbers (%) and compared between treatment and failure groups.

Variable	Total (n = 249)	Successful intervention (n = 184)	Treatment failure (n = 65)	p
Female	103 (41.4%)	76 (41.3%)	27 (41.5%)	0.974
Age (years)	62.17 (19.34)	60.92 (20.2)	65.71 (16.1)	0.057
BMI (kg/m^2^)	26.58 (5.16)	26.41 (4.8)	27.03 (5.9)	0.429
Duration of symptoms (days)	7.18 (7.85)	5.84 (6.4)	11.34 (10.3)	**0.003**
Fever at admission	53 (21.28%)	42 (22.8%)	11 (16.9%)	0.318
Diabetes	62 (24.90%)	44 (23.9%)	18 (27.7%)	0.545
Insulin dependent diabetes	21 (8.43%)	12 (6.5%)	9 (13.8%)	0.068
Chronic renal insufficiency	55 (22.09%)	39 (21.2%)	16 (24.6%)	0.568
HIV	2 (0.80%)	2 (1.1%)	0 (0.0%)	> 0.999
Rheumatoid arthritis	17 (6.83%)	12 (6.5%)	5 (10.8%)	0.748
Hepatitis C	7 (2.81%)	5 (2.7%)	2 (3.1%)	> 0.999
Alcohol abuse	22 (8.84%)	19 (10.3%)	3 (4.6%)	0.208
IV drug abuse	6 (2.41%)	5 (2.7%)	1 (1.5%)	> 0.999
CCI	3.52 (2.64)	3.30 (2.72)	4.14 (2.33)	**0.027**
**Kellgren and Lawrence**				
0	19 (9.69%)	16 (11.7%)	3 (5.1%)	0.193
1	31 (15.82%)	25 (18.2%)	6 (10.2%)	0.155
2	59 (30.10%)	44 (32.1%)	15 (25.4%)	0.349
3	52 (26.53%)	33 (24.1%)	19 (32.2%)	0.347
4	36 (18.37%)	19 (13.9%)	17 (28.8%)	**0.013**
**Gächter**				
1	24 (23.30%)	29 (23.2%)	5 (23.8%)	0.951
2	49 (47.57%)	39 (47.6%)	10 (47.6%)	0.996
3	26 (25.24%)	20 (24.4%)	6 (28.6%)	0.694
4	4 (3.88%)	4 (4.9%)	0 (0.0%)	0.579
**Surgery**				
Duration knee (min)	52.70 (21.97)	53.02 (23.96)	51.81 (14.90%)	0.701
Duration shoulder (min)	48.72 (26.94)	48.97 (30.02)	48.08 (17.39%)	0.920
Arthroscopy knee	140 (71.87%)	109 (75.2%)	31 (63.3%)	0.108
Arthroscopy shoulder	20 (36.36%)	10 (26.5%)	10 (62.5%)	0.010

Values in bold indicate statistically significant results.

**Table 2:** Weights used for the predictive score based on respective relative risks. 0–22 SYNC3 points can be reached by addition of respective weights on day three after surgery. If CRP levels decrease less than 2 mg/dl within five days, another four points are added, resulting in the SYNC5 score.

Variable	Weight
Symptom duration > 4 days	5
CCI ≥ 3	4
Positive culture results	5
CRP reduction by day three post OP < 0.7 mg/dl	3
Neutrophilic granulocyte reduction by day three post OP < 2.3 G/l	5
CRP reduction by day five post OP < 2 mg/dl	4

### Treatment

Patients underwent joint punctures at our emergency department at initial presentation, two samples were sent for microbiological analysis. Antibiotic therapy with Cefazolin was initiated immediately after joint aspiration, and in case of penicillin allergy clindamycin was administered instead. Surgery was performed within 24 h, and at least three more tissue as well as fluid samples were taken during surgery and sent for histological and microbiological analysis. As soon as culture and antibiogram results were available, therapy was adjusted in consultation with the department of infectious diseases. Two weeks of i.v. antibiotics were followed by four additional weeks of oral administration.

The decision for arthroscopy or open arthrotomy was left to the executing consultant orthopedic surgeon. For the shoulder arthrotomy, an anterior deltopectoral approach with subscapularis tenotomy was the method of choice. For the shoulder arthroscopy, either three standard portals (posterior, anterosuperior, and lateral portal) or four standard portals (posterior, anterosuperior, anterolateral, and posterolateral portal) were used. For knee arthrotomy, a medial parapatellar approach was chosen. In case of knee arthroscopy either three (anterolateral, anteromedial, and superomedial) or four portals (anterolateral, anteromedial, superomedial, superolateral) were used.

All surgical procedures included synovectomy, debridement, and joint irrigation with sterile physiological sodium saline solution to an extent determined by the surgeon, as well as microbiological and histopathological sampling. No local antibiotics were administered intraoperatively. One or two suction drains were routinely employed per joint and usually removed five days postoperatively.

Treatment failure was defined as the need for more than one surgical procedure for infection eradication, and suspected in case of elevated inflammatory markers, joint pain, limited range of motion or purulent swelling. In case of suspected failure, patients underwent another surgery within less than 48 h. Patients underwent examinations and blood testing in an outpatient setting at two and six weeks, and at three, six and twelve months after surgery, and were readmitted and underwent another debridement in case of suspected reinfection. The total median follow-up time was 32 months, with 25%- and 75%-percentiles at 5.8 and 70.0 months respectively. This study was approved by the ethics committee of the Medical University of Vienna (identification number 1296/2019). All research was performed in accordance with relevant guidelines and regulations, and informed consent was obtained from all patients.

### Statistics

Continuous variables were described using mean and standard deviation and compared via t-tests, or non-parametrical Mann–Whitney-U testing if any conditions for the t-test were not fulfilled. Binary and categorical variables were described via percentages and compared by Chi-squared testing, or Fischer’s exact test for less than five observations.

In terms of model development, we first chose all significant demographic variables from univariate analysis, except for Kellgren-Lawrence classifications and surgery types due to a lack of data. Laboratory parameters whose postoperative decline differed significantly between groups were additionally included. All continuous variables used for score development were transformed to dichotomous variables by calculating cutoffs based on the respective Youden index. All variables were then weighted based on their relative risk for failure of a single irrigation and debridement. We assessed performance via receiver operating characteristic (ROC) curves. The Cochran-Armitage trend test was used to evaluate the correlation between score values and the need for repeated surgery.

All tests were calculated in their two-sided version with a p-value of < 0.05 considered as significant. The analysis was performed using SPSS Version 28 (IBM Corp., Armonk, NY, USA).

## Results

### Univariate analysis

**Figure 2:**
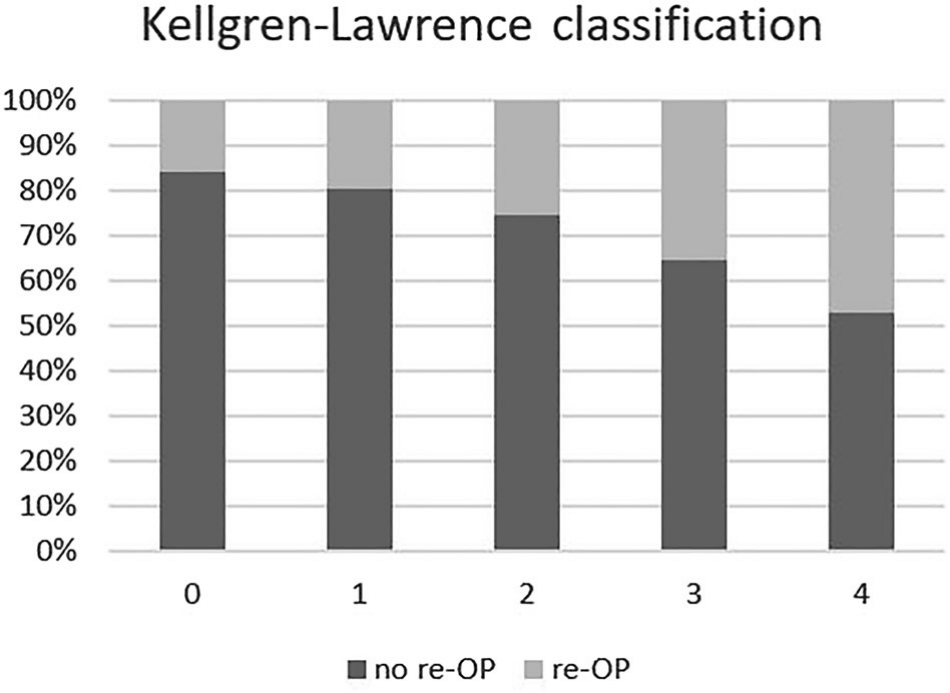
Distribution of Kellgren-Lawrence classes in the success and failure group.

249 patients with septic monoarthritis, including 194 knee (77.9%) and 55 shoulder joints (22.1%) were analyzed in total. Sixty-five (26.1%) experienced failure of a single surgical debridement (25.3% of shoulders, 29.1% of knees). Median follow-up for the single-surgery group was 34 months, and 21 for the failed single-surgery group, respectively. Median time to second surgery was 15 days. Infectious etiology could be retraced in 141 patients, including 61 iatrogenic (56.5%), 46 hematogenic (42.6%), and one traumatic case (1.2%). There were no significant differences regarding gender, age, BMI, fever at admission, Gächter score, and surgery duration between successful and failed single surgical debridement (p > 0.05). Higher CCI scores were significantly associated with the need for repeated surgery (p = 0.025). Further, longer symptom duration (p = 0.003) and severe osteoarthritis, as reflected by high grades of the Kellgren-Lawrence classification (cf. Figure 2), were significantly associated with failure of a single debridement (p = 0.013). Table 1 summarizes the results in detail.

Of the 65 patients experiencing treatment failure, 39 (60%) were successfully reoperated, 18 (27.7%) required further surgical interventions for septic arthritis, 4 (6.2%) presented with impaired wound healing, 3 (4.6%) died in the postoperative course, 2 (3.1%) were admitted to the ICU for septic shock. One (1.5%) knee arthrodesis was performed.

### Surgical technique

In total 89 arthrotomies (35.7%; 35 shoulders, 54 knees) and 160 arthroscopies (64.3%; 20 shoulders, 140 knees) were performed. The surgical technique had a significant impact on shoulder joints, with failure rates of 50.0% for arthroscopy compared to 17.1% for arthrotomy (p = 0.01). The comparison of baseline characteristics between both treatment strategies showed a significantly higher percentage of female patients (50.6% vs. 36.2%, p = 0.04), a higher mean age (67.0 vs. 59.3 years, p = 0.003), as well as a higher CCI in the arthrotomy group (4.28 vs. 3.09, p < 0.001). Arthroscopy was performed more frequently in knee joints (60.7 vs. 87.5%, p < 0.001), and average Gächter scores were higher in the arthrotomy group (p = 0.011).

### Microbiological analysis

**Figure 3:**
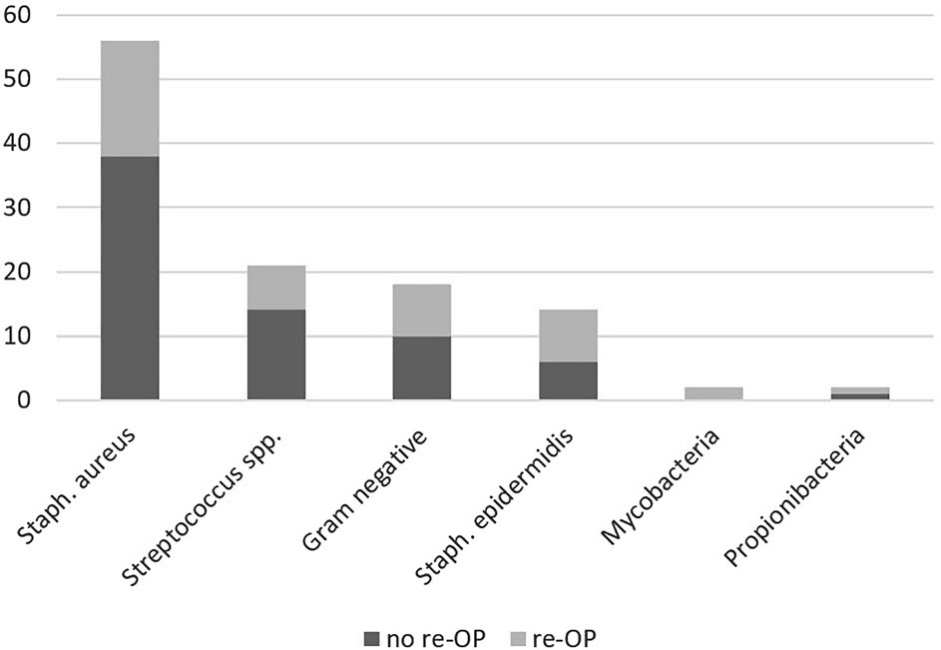
Distribution of pathogens over a total of 112 positive bacterial cultures.

Positive cultures were found in 37.9% in the successful single debridement group, and in 66.15% in the failure group respectively (p < 0.01). In patients in whom no pathogens could be detected, the diagnosis was confirmed by intraoperative histopathological examination. *Staphylococcus aureus* infections showed no significant influence on treatment failure (p = 0.187). Methicillin-resistant *Staphylococcus aureus* (MRSA) infections were not separately analyzed, as there were too few cases (cf. Figure 3).

### Postoperative laboratory parameters

**Figure 4:**
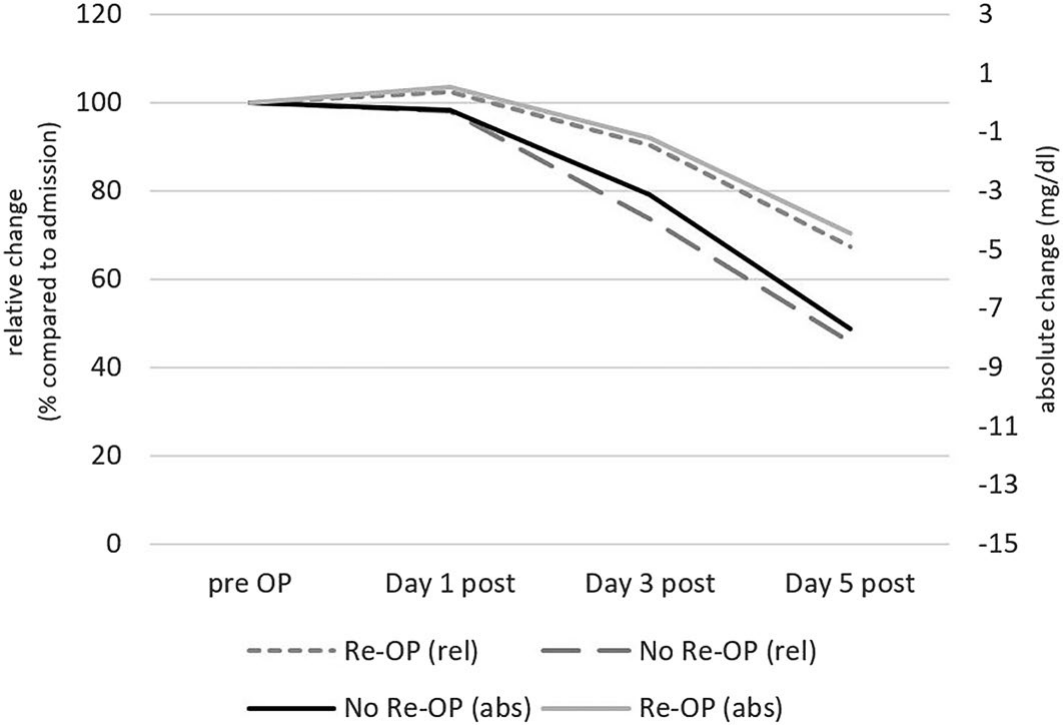
Absolute (abs) and relative (rel) CRP changes compared to admission values.

CRP values on the third and fifth postoperative day were significantly higher in the treatment failure group. Neutrophilic granulocytes three days after surgery, and hemoglobin levels before discharge showed a significant difference in absolute as well as relative measures (cf. Table 3 and Fig. 4).

**Table 3:** Laboratory values described by mean (SD) and compared between successful treatment and failure groups.

Variable	Total (n = 249)	Successful intervention (n = 184)	Treatment failure (n = 65)	p
**HbA1c at admission (%)**	6.22 (1.46)	6.21 (1.45)	6.22 (1.49)	0.961
**Creatinine**				
At admission	1.18 (0.81)	1.18 (0.86)	1.17 (0.69)	0.906
Day 3 to 5 post OP	1.03 (0.77)	1.03 (0.81)	1.04 (0.65)	0.881
Before discharge/reoperation	0.97 (0.58)	0.98 (0.64)	0.95 (0.37)	0.708
**CRP (mg/dl)**				
At admission	15.83 (10.35)	15.56 (10.36)	16.58 (10.38)	0.498
Day 1 postoperative	15.81 (9.86)	15.23 (9.69)	17.29 (19.24)	0.190
Day 3 postoperative	12.60 (8.85)	11.27 (7.78)	15.58 (9.84)	**0.002**
Day 5 postoperative	9.24 (7.99)	7.71 (6.45)	13.24 (10.07)	** < 0.001**
Day 1 post OP—Admission	−0.37 (7.54)	−0.59 (8.09)	0.19 (5.96)	0.644
Day 3 post OP—Admission	−3.91 (10.01)	−4.84 (9.89)	−1.55 (10.02)	**0.032**
Day 5 post OP—Admission	−7.17 (10.41)	−8.26 (9.87)	−4.31 (11.31)	**0.015**
**WBC (G/l)**				
At admission	11.81 (4.70)	11.91 (4.89)	11.49 (4.16)	0.528
Day 1 postoperative	9.71 (4.09)	9.70 (4.36)	9.74 (3.32)	0.953
Day 3 postoperative	8.71 (4.17)	8.79 (4.41)	8.51 (3.48)	0.664
Day 5 postoperative	8.78 (4.17)	8.79 (3.82)	8.74 (3.28)	0.927
In punctate	66.36 (50.59)	70.60 (52.64)	45.16 (34.64)	0.267
**Neutrophil granulocytes (G/l)**				
At admission	7.83 (3.08)	7.99 (3.27)	7.67 (2.50)	0.711
Day 3 postoperative	6.17 (2.27)	5.95 (2.19)	6.80 (2.40)	**0.019**
Day 3 post OP—Admission	−1.89 (2.29)	−2.19 (2.35)	−1.03 (1.87)	**0.008**
**Haemoglobin (g/dl)**				
At admission	12.38 (2.00)	12.48 (2.03)	12.09 (1.91)	0.179
Day 3 to 5 post OP	11.29 (1.77)	11.41 (1.84)	10.97 (1.52)	0.086
Last before discharge or reoperation	11.24 (1.70)	11.52 (1.67)	10.54 (1.58)	** < 0.001**
Day 3 to 5 post OP–Admission	−13.31 (3.14)	−1.03 (1.32)	−1.15 (1.54)	0.504
Last before discharge or reoperation—Admission	−1.02 (1.53)	−0.82 (1.47)	−1.48 (1.57)	** < 0.001**
**Fibrinogen (mg/dl)**				
At admission	737.54 (215.44)	740.96 (216.97)	729.44 (213.29)	0.725
Day 1 postoperative	658.88 (169.98)	646.45 (150.51)	683.98 (203.21)	0.212
Day 3 postoperative	688.70 (187.08)	681.63 (167.18)	703.71 (224.75)	0.542
Day 5 postoperative	644.71 (200.45)	633.76 (187.80)	669.14 (242.52)	0.338
**Microbiology**				
Positive culture	112 (44.98%)	69 (37.91%)	43 (66.15%)	** < 0.001**
Staph. aureus	55 (48.67%)	37 (53.62%)	18 (40.91%)	0.187

Values in bold indicate statistically significant results.

### Score development

**Figure 5:**
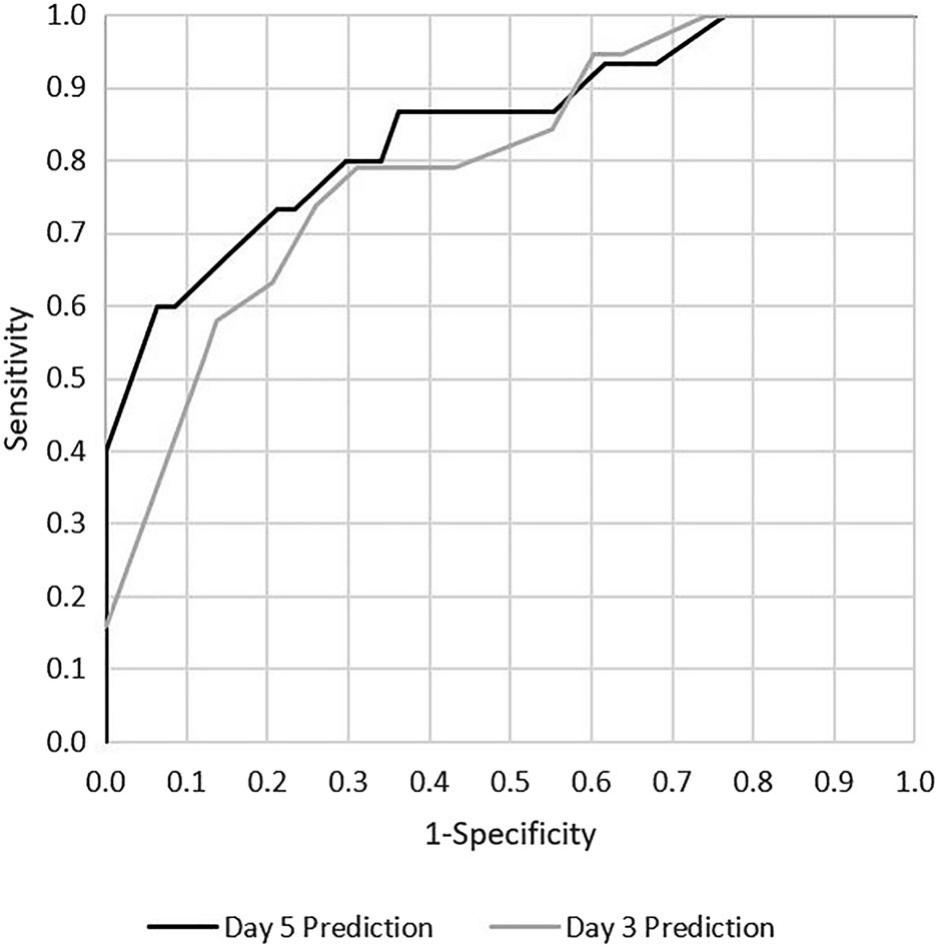
ROCs from the two prediction models. The first includes information on CRP and neutrophilic granulocytes up to day 3 post surgery, symptom duration, CCI, and bacterial culture result. The second model additionally includes information on CRP change from admission to day 5 post-OP.

**Figure 6:**
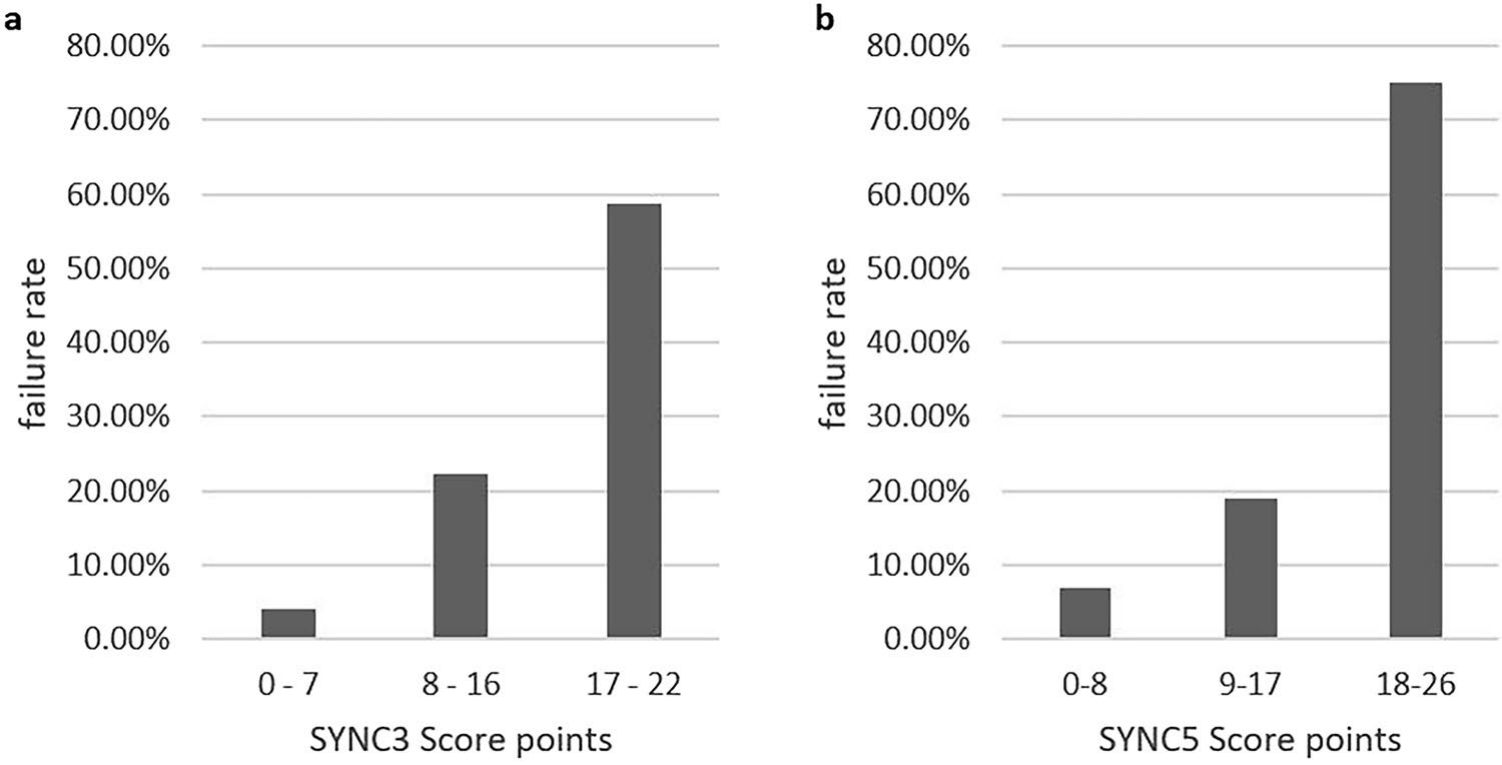
Low, intermediate, and high risk of failure depending on score points on the 3rd and 5th postoperative day.

Two different SYNC (Symptom duration, Neutrophilic granulocytes, Charlson comorbidity index, 3rd day CRP, 5th day CRP) scoring systems were developed: the SYNC3 includes influencing factors that are known as early as day 3 after surgery, the SYNC5 is a modified version additionally including CRP drop between admission and the fifth postoperative day. Calculated weights for each of the variables are given in Table 2. The first score reached an AUC of 0.80 (p < 0.001, CI = [0.69 0.91]), the second had an AUC of 0.845 (p < 0.001, CI = [0.72 0.97]) , and both can be considered as excellent in terms of discrimination. Corresponding ROCs are shown in Fig. 5. Positive correlation of risk score categories and failure rates was underlined by the Cochran-Armitage trend test with p < 0.0001 for both scores. Patients were grouped into low, intermediate, and high risk of failure based on their score results (cf. Figure 6). Additionally, a website for score calculation was created (www.ortho-score.com).

### Discussion

Septic arthritis of large joints is potentially life-threatening, and requires repeated intervention if a single irrigation and debridement was not sufficient to control the infection to prevent further joint destruction^19^. To the best of our knowledge, we evaluated the largest patient cohort on risk factors for failure of a single arthroscopy or arthrotomy for septic arthritis to date^19–21,23^. A new scoring system for treatment failure risk estimation was created based on symptom duration, CCI, bacterial culture results, postoperative change in CRP, and change in neutrophilic granulocytes.

This is the first study to demonstrate that higher postoperative CRP and neutrophilic granulocyte levels only a few days after initial surgical intervention, as well as slow decrease of absolute and relative CRP and neutrophile values are significant risk factors for failure of a single surgical intervention. The direct proportionality of absolute and relative changes in this study highlight the known physiological exponential course of CRP and neutrophil levels^27,28^. Consequently, it was sufficient to include one of the two progression parameters into the SYNC scores. The predictions were based on symptom duration, CCI, bacterial culture results, and absolute CRP and neutrophilic granulocyte changes by the third postoperative day, achieving an AUC of 0.80. Including additional information on the CRP change from admission to the fifth postoperative day further increased the AUC to an excellent 0.85. However, as there was not enough data available for internal validation, external validation is required before our scoring systems can be used in clinical practice.

In our cohort 65 patients (26%) had a suspected reinfection with consequent need for further surgical treatment. These numbers are comparable to recent studies, which described rates between 11 and 40%^2,3,7,18–23^. In terms of surgical technique, arthroscopy was the preferred method in case of knee infection (72%) and arthrotomy was chosen for the majority of shoulder infections (64%). No significant differences regarding failure rates were observed for knee infections. Though, we found a trend towards better results after arthroscopy, which is consistent with current literature, indicating that knee arthroscopy reduces reoperation rates while improving postoperative range of motion^2^. Shoulder arthroscopy was associated with a higher failure rate in our study, yet current evidence does not provide a clear indication of the superiority of one surgical method over the another. Failure rates in current literature range from 5.6 to 100%, and Abdelmalek et al. found higher reoperation rates in arthroscopy in comparison to arthrotomy for the treatment of native shoulder septic arthritis, Acosta-Olivo et al. report a tendency towards lower reinfection rates for arthroscopy, and Memon et al. did not demonstrate the superiority of either method^3,7,12,18,29,30^.

We observed a trend towards lower Gächter classes in patients treated with arthroscopy compared to arthrotomy, but no difference in Gächter stages regarding the risk of failure. This underlines previous findings, indicating that patients with a higher Gächter classification are more likely to benefit from arthrotomy than from arthroscopy, and appropriate choice of surgical procedure can help minimize the risk of failure^31^.

Our study yielded positive microbiological cultures of joint aspirate for almost half of all cases, twice as frequent and therefore significantly more common in the failure group, in which infection was confirmed histologically. The current body of evidence highlights that negative microbiological cultures occur in 12–50% of cases despite strong clinical suspicion of septic arthritis, especially if crystals or clotting are present^20–22,32^. The sensitivity of bacterial culture reportedly ranges between 75–95%, which might decrease in case of atypical organisms or antibiotic treatment before joint aspiration, and in 9–14% of cases the pathogen can only be detected through additional blood cultures^33,34^. This underlines the necessity for a more comprehensive approach to diagnosis including clinical, radiological, and histological evidence^2,35^.

In the presented cohort, *Staphylococcus aureus* was the most common pathogen and accounted for 49% of all organisms, similar to previous reports ranging between 37–56%^2,19,36–38^. Patients with *Staphylococcus aureus* infections showed no significantly higher failure rate in our study. However, current data on this topic is ambiguous: some studies found a significant association^12,19,24^, whilst others did not^7,20^. Different rates of MRSA infections might explain the contradicting findings. In our cohort, there were only four MRSA cases, and therefore too few to draw conclusions and far fewer than reported in comparable studies^7,19,39^. Geographical differences in the presence of MRSA might explain the inconsistent findings^40^.

The current study confirms previous observations that symptom duration and CCI are risk factors for failure of a single surgical intervention. However, none of the analyzed single comorbidities was associated with intervention failure^3,7,22^. Further, we found a significant difference of about 1 mg/dl in Hb levels at discharge or before reoperation between successful and failed single surgical intervention groups. This might be explained though a more radical synovectomy, and therefor increased blood loss, for patients suffering from a more severe infection, but a more detailed assessment of postoperative Hb levels is needed.

As of today, there exists no guideline on how to decide whether or when to repeat irrigation and debridement in patients with suspected persistent infection, and there is currently no uniform definition of treatment failure^7^.

The median time to second surgery was 15 days within our cohort, but for the majority of patients classified as high risk, repeated surgery was correctly predicted 10 days earlier than that. This indicates that levels and changes of infection parameters only a few days after initial surgery already need to be considered as risk factors for treatment failure, instead of drawing conclusions from them only after more extended periods of watchful waiting. Therefore, a faster decision for return to operating room, reinforced by score results, could potentially prevent further joint destruction, and preserve functionality. As for patients in the intermediate risk group, the indication for earlier and more frequent follow-ups may be made.

The current study has some limitations: First, although it is based on the largest dataset of risk factors for shoulder and knee arthritides in current literature, its sample size still is comparatively small. Including the two most commonly affected large joints on the one hand increased sample size, but on the other hand neglect joint specific pathophysiology or treatment response. Our data quality was also limited by the retrospective data collection, with consecutive incompleteness and missing randomisation. No functional parameters could be considered due to insufficient documentation, and information on the administration of antibiotics prior to hospital admission was not available. Second, as there were not enough data for internal validation, there is a clear risk of overfitting for our predictive scores, which needs to be taken into consideration when applying them in clinical practice. Third, in terms of surgical approaches, decisions were made based on the opinion of the consultant orthopedic surgeon.

In summary, the course of CRP and neutrophil granulocytes between admission and the fifth postoperative day, in combination with CCI, symptom duration, and bacterial culture results can help predict the need for further surgical intervention earlier and more accurately. Moreover, a decrease in hemoglobin and high Kellgren-Lawrence grading as factors significantly associated with more than one surgery.

## Data Availability

The datasets generated and/or analyzed during the current study are not publicly available, as participants of this study did not agree for their data to be shared publicly. They are available from the corresponding author on reasonable request, after obtaining permission from the ethics board of the Medical University of Vienna.
